# Post-orgasmic Illness Syndrome: A Case Report

**DOI:** 10.7759/cureus.61494

**Published:** 2024-06-01

**Authors:** Fahad K Alanazi, Abdullah Ismael Sawma, Moferah Harisi, Meshal Albabtain, Ali Almuzaini

**Affiliations:** 1 Urology, King Saud Medical City, Riyadh, SAU

**Keywords:** fever, urology, flu-like symptom, pois, post-orgasmic illness syndrome

## Abstract

Post-orgasmic illness syndrome (POIS) is a rare condition characterized by debilitating symptoms following ejaculation. We present a case of a 25-year-old male with flu-like symptoms post-ejaculation since age 17. Despite minimal relief from conventional treatments, a comprehensive evaluation led to the diagnosis of POIS and successful management with niacinamide therapy. The presentation of flu-like symptoms following ejaculation in this case raises several questions regarding the underlying pathophysiology. While the exact cause of his symptoms remains elusive, the resolution achieved with niacinamide therapy underscores the importance of considering alternative treatment modalities in complex cases. The role of varicocele in symptom manifestation, if any, also warrants consideration, as varicocele has been associated with male infertility and testicular dysfunction.

## Introduction

In the realm of urology, a myriad of symptoms and conditions present themselves, often challenging both patients and healthcare providers. Post-orgasmic illness syndrome (POIS) is a rare and poorly understood condition characterized by a constellation of physical and psychological symptoms following ejaculation. Individuals with POIS experience a range of debilitating effects that can persist for days, significantly impacting their quality of life. Despite its limited recognition in the medical community, POIS poses significant challenges in diagnosis and management [1].

Symptoms of POIS typically begin within minutes to hours after orgasm and can include fatigue, muscle aches, headaches, cognitive difficulties, and flu-like symptoms such as fever and chills. In some cases, individuals may also experience emotional symptoms such as irritability, anxiety, and depression. These symptoms can be severe enough to interfere with daily activities and relationships, leading to feelings of frustration and isolation [1].

Diagnosing POIS can be challenging, as the condition is not well known among healthcare providers and there are no specific tests to confirm its presence. However, a thorough medical history and physical examination can help rule out other potential causes of symptoms. Keeping a detailed diary of symptoms and their timing concerning sexual activity can also help make a diagnosis [2].

Treatment options for POIS are limited and often focus on managing symptoms rather than addressing the underlying cause of the condition. Antihistamines, nonsteroidal anti-inflammatory drugs (NSAIDs), and corticosteroids may be prescribed to help alleviate symptoms such as inflammation and pain. Lifestyle modifications such as avoiding certain foods or activities that may trigger symptoms, practicing stress-reducing techniques, and maintaining a healthy diet and exercise routine may also be recommended [1,2].

## Case presentation

A 25-year-old male presented to the urology clinic with the chief complaint of flu-like symptoms, experiencing a range of symptoms post-ejaculation, including exhaustion, fatigue, general malaise, dry eyes, painful muscles and knees, and heaviness in the legs. These symptoms manifested approximately an hour after ejaculation and persisted for a week, significantly impacting his quality of life. Despite seeking help from a psychiatrist at another hospital and being prescribed escitalopram 10 mg and Voltaren 100 mg, the patient experienced only minimal relief.

Upon physical examination, the patient appeared healthy, with normal male facial distribution. Examination of the genitalia revealed normal findings, except for the presence of a grade 2 left varicocele (Table 1).

**Table 1:** Patient laboratory findings with normal values included LH: luteinizing hormone

Laboratory finding	Patient value	Normal range
LH	5.93 mIU/mL	1.7-8.6 mIU/mL
Estradiol	103 pg/mL	27-122 pg/mL
Prolactin	9.16 ng/mL	2.1-17.7 ng/mL
Testosterone	22.53 nmol/L	9.9-27.8 nmol/L

Following the comprehensive evaluation, the patient was initiated on niacinamide therapy (500 mg per day, taken orally in the form of a tablet) for five months. Niacinamide, a form of vitamin B3, is known for its role in cellular energy production and overall metabolic function. Its mechanism of action in alleviating the post-ejaculation symptoms in this case remains an area of interest and warrants further exploration.

After the initiation of niacinamide therapy and the observed improvement in the patient's symptoms, a structured follow-up plan was established to monitor treatment response and adjust therapy as needed. This follow-up plan included regular check-ins with the healthcare provider to assess symptom severity, discuss any changes in the patient's condition, and evaluate the overall effectiveness of the treatment to provide ongoing support and improve the patient's quality of life. Regular follow-up visits were scheduled to assess symptom progression, evaluate the varicocele's response to treatment, such as decreased discomfort and improved quality of life, and address any emerging concerns or side effects. Close collaboration between the urology, endocrinology, and nutrition teams was maintained to ensure coordinated care and optimize the patient's overall well-being. 

The patient was also followed up in the psychiatric clinic. Psychiatric support included psychoeducation to help the patient understand the condition's symptoms and treatment options. Cognitive behavioral therapy (CBT) was utilized to identify and challenge negative thoughts and behaviors related to POIS and develop healthier coping strategies. The patient was taught mindfulness and relaxation techniques to reduce anxiety and improve mental well-being. Additionally, connecting the patient with support groups provided a sense of community and validation in a safe environment. Lifestyle recommendations focused on regular physical activity, a balanced diet, and healthy habits to support mental health. Regular follow-up appointments were scheduled to monitor the patient's progress and make any necessary adjustments to the treatment plan.

Overall, the psychiatric support plan aimed to address the psychological and emotional impact of POIS on the patient's well-being. By providing a holistic approach that included education, therapy, mindfulness practices, medication management, lifestyle recommendations, and ongoing monitoring, healthcare providers sought to support the patients in managing their condition and improving their mental health outcomes.

The long-term prognosis for the patient hinges on various factors, including treatment adherence, response to therapy, and the presence of any underlying conditions that may influence symptom recurrence. The comprehensive evaluation, multidisciplinary management, and tailored follow-up plan were essential components in addressing the patient's complex presentation and working toward a favorable outcome.

## Discussion

POIS is a rare condition that affects individuals shortly after orgasm. It is characterized by a range of physical and psychological symptoms that can last for several days, significantly impacting the quality of life of those affected. While the exact cause of POIS is not fully understood, researchers believe that it may be related to an allergic or inflammatory response to substances released during ejaculation [1,3].

The initial study on POIS described two men who experienced rapid-onset flu-like symptoms, including feverishness, extreme fatigue, burning eyes, cognitive disturbances, and irritability following ejaculation. These symptoms persisted for three to five days and recurred with the same intensity upon subsequent ejaculations, leading the individuals to abstain from sexual activity to avoid illness. Despite being in good physical health otherwise, the immediate onset of symptoms after ejaculation led researchers to speculate that POIS might be triggered by an immunological process, given the rapid and severe nature of the physical and mental manifestations [4].

The hypothesis suggested by Waldinger and Schweitzer proposed that the immune system might play a central role in the development of POIS, as only the immune system could induce such swift and profound symptoms. Importantly, they suggested that the immune response was systemic rather than localized to the genital area. This theory found support in existing literature on cytokines, which are known to induce flu-like symptoms. However, the specific role of cytokines in mediating POIS was not yet understood at that time, highlighting the need for further research to elucidate the immunological mechanisms underlying this enigmatic condition [5].

Research into POIS is ongoing, and there is still much to learn about this complex and poorly understood condition. Studies have suggested that POIS may be more common than previously thought, with some estimates suggesting that up to one in 100 men may be affected. There have been fewer than 50 cases recorded in the literature since POIS was first described [6,7].

More awareness and understanding of POIS among healthcare providers and the general public are needed to improve diagnosis and treatment options for those affected.

## Conclusions

The case serves as a poignant reminder of the diverse and enigmatic nature of urological conditions. Through a meticulous evaluation encompassing physical examination, laboratory investigations, and targeted therapy, healthcare providers can offer effective solutions to patients grappling with such perplexing symptoms. Continued research and clinical vigilance are pivotal in unraveling the mysteries surrounding rare presentations like this, ultimately enhancing patient care and outcomes in the field of urology.

This case exemplifies the intricate interplay between clinical acumen, investigative prowess, and therapeutic innovation in managing complex urological presentations, shedding light on the importance of a holistic approach to patient care in the ever-evolving landscape of medicine.
